# Poultry Preslaughter Operations in Hot Environments: The Present Knowledge and the Next Steps Forward

**DOI:** 10.3390/ani14192865

**Published:** 2024-10-04

**Authors:** Frederico Márcio Corrêa Vieira, Miguel Angel Guillen Portugal, Leonardo Piffer de Borba, Sabina Angrecka, Piotr Herbut, Ayoola Olawole Jongbo, Karolini Tenffen De-Sousa, Matheus Deniz

**Affiliations:** 1 Biometeorology Study Group (GEBIOMET), Universidade Tecnológica Federal do Paraná (UTFPR), Estrada para Boa Esperança, km 04, Comunidade São Cristóvão, Dois Vizinhos 85660-000, Paraná, Brazil; miguelguillenportugalvet@gmail.com (M.A.G.P.); leopborba@yahoo.com (L.P.d.B.); sabina.angrecka@urk.edu.pl (S.A.); piotr.herbut@urk.edu.pl (P.H.); aojongbo@gmail.com (A.O.J.); karoltenffen10@hotmail.com (K.T.D.-S.); m.deniz@unesp.br (M.D.); 2 Department of Rural Building, Faculty of Environmental Engineering and Land Surveying, University of Agriculture in Krakow, 31-120 Kraków, Poland; 3 Department of Agricultural and Environmental Engineering, School of Engineering and Engineering Technology, Federal University of Technology, PMB 704, Akure 340110, Ondo State, Nigeria; 4 School of Veterinary Medicine and Animal Science—São Paulo State University, Botucatu 18618-681, São Paulo, Brazil

**Keywords:** preslaughter operations, broiler, animal welfare, food safety, logistics

## Abstract

**Simple Summary:**

The poultry meat industry is a significant agri-food sector worldwide. Preslaughter operations are crucial as they impact animal welfare, meat quality, and food safety. Despite their importance, few studies focus on broiler preslaughter processes in hot environments. Effective planning, handling, and monitoring are essential to improve conditions and reduce stress, ensuring better meat quality and animal welfare.

**Abstract:**

Poultry production faces significant challenges, including high feed prices, diseases, and thermal stress, which impact broiler welfare and productivity. Despite advances in cooling technologies and ventilation, preslaughter operations still lead to considerable losses. This review highlights the need for the improved management of thermal environments and animal logistics. Preslaughter operations typically involve fasting broilers for 8–12 h to reduce gastrointestinal contents and contamination. Following fasting, broilers are caught, crated, and transported. Stress levels vary based on distance and conditions, with manual catching often causing stress and injuries. Catching should occur during cooler periods to minimise these issues, and transport conditions must be carefully managed. Lairage, the waiting period after transport, should be kept short (1–2 h) in climate-controlled environments to avoid stress and deterioration. Proper handling and efficient unloading are essential to prevent injuries and reduce economic losses. Stunning methods, such as electronarcosis and a controlled atmosphere, aim to minimise suffering before slaughter, though practices vary culturally and religiously. Logistics and real-time monitoring technology are crucial for enhancing animal welfare during transportation. Effective planning and the optimisation of transport processes is vital for reducing stress and losses, especially with regard to rising global temperatures and production demands.

## 1. Introduction

Nowadays, many factors, such as the high feed price oscillation, the current challenges regarding bird diseases, and harsh environmental conditions, are factors that impair poultry production. Those animals are limited physiologically regarding thermal transfer possibilities due to their feather covering, increased metabolic rate, and high rearing density in poultry housing. Also, the increase in air temperature and relative humidity causes severe thermal stress in birds, which continues to be a significant limiting factor in poultry production [1,2]. In addition, a high thermal load could result in a lower feed intake, decreased lipid utilisation, altered glucose homeostasis, and increased fat deposition and oxidation stress [3]. Over time, the thermal load has been significantly reduced with ventilation, advanced cooling technologies, intelligent tools to estimate heat stress, and the supplementation of antioxidants for the homeostatic regulation of birds within the aviary [4,5,6]. However, once the broilers’ slaughter weight is met, the lack of control over external factors at preslaughter operations remains the leading cause of economic losses due to mortality, injured animals, and the loss of carcass quality [7]. Kittelsen et al. [8] found a higher rate of sudden death (57.2%) among broilers who were dead on arrival. This result was explained by acute stress during the preslaughter operation, mainly during catching, crating, and transport, which caused lung congestion. Additionally, when the broilers were manually loaded, the loading duration was higher than that of mechanical loading, which increased the injury risk (wing hematomas) over time [9].

During the preslaughter operations, the birds are exposed to different management and stress processes until they reach the slaughter line, starting with the removal of food (fasting), catching, loading, transport, and lairage at the slaughterhouse, which, both uniquely and cumulatively, can contribute to losses and poor animal welfare [10,11,12]. The management procedure, from bird catching to the arrival at slaughterhouses, is often managed by industry protocols with little or no scientific background, which increases the threat of stress and the poor welfare of these animals. Thus, the concept and scientific basis of animal logistics needs to be addressed. Based on this, we aimed to review broilers’ thermal stress and welfare concerning preslaughter operations in hot environments. The following topics regarding the preslaughter operations will be discussed: the main thermal stress signals, fasting, broiler catch or capture, loading, transportation, lairage, and the slaughter of the animal. Finally, we discussed some perspectives regarding the following steps to advance the understanding and promotion of animal logistics practice for broiler chickens.

## 2. A Brief Synthesis of the Thermal Stress of Poultry

The body temperature of adult birds fluctuates between 41 and 42 °C [13]. Heat is sensibly transferred from birds to the environment by radiation, convection, and conduction during fluctuations in the environmental temperature. When this temperature rises to 42–42.5 °C, the birds start panting, or thermal polypnea is triggered [14]. Prolonged panting leads to the excessive loss of CO_2_ from the body, resulting in respiratory alkalosis [15]. The birds also adopt unusual physical postures and behavioural strategies to help heat loss, e.g., by extending the neck while standing, holding the wings away from the body, and vasodilatation in the naked parts of the body to dissipate heat [16]. During low ambient temperatures, the birds increase their catabolic rate to prevent a sudden fall in the core body temperature. When the environmental temperature falls, the birds stick together and fluff out their feathers to increase the effective surface area. Further, the birds sit and tuck their heads under the wing to decrease heat loss from their unfeathered portions [17].

During exposure to high environmental temperatures, peripheral thermal receptors are stimulated, suppressing the appetite centre in the hypothalamus, leading to reduced feed intake [18]. The metabolic rate is boosted by triiodothyronine (T3), and under heat stress, the concentration of T3 is depressed, and thyroxin (T4) is increased [19]. The altered concentrations of T3 and T4, the reduced enzymatic activities of chymotrypsin, trypsin and amylase, and a decreased feed intake at high environmental temperatures may depress body weight gain [20,21]. Protein biosynthesis is also adversely affected at high ambient temperatures, impairing metabolism. Another problem with broilers reared under high ambient temperature is a heavier body weight and a lower carcass quality.

Excessive fat in broilers is a well-known public concern. In broilers, fat deposition is increased under high ambient temperatures [22]. Heat stress affects carcass composition and meat yield, mainly of breast meat [23]. Broilers reared under high-temperature environments have less breast, thigh, and drumstick weight [24], possibly due to the overproduction of corticosterone [25].

Further, with the reduction in breast weight, there is a decrease in the protein content due to a reduction in the synthesis of muscle protein, accelerated protein breakdown, and an increase in fat content due to reduced basal metabolism and physical activity [23]. Intramuscular, subcutaneous, and abdominal fat deposits are enhanced in broilers at high environmental temperatures [22].

## 3. The Poultry’s Preslaughter Operations

**Figure 1:**
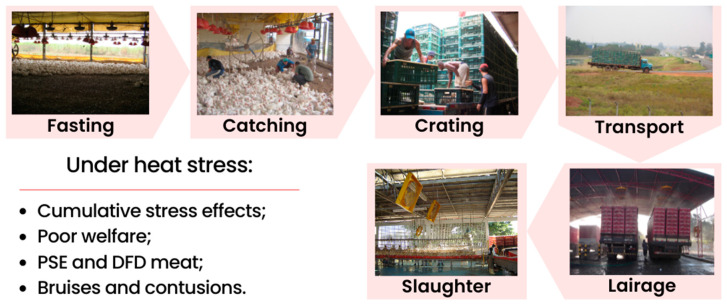
Schematic diagram of the poultry preslaughter steps.

The preslaughter operations begin with the fasting of broilers for 8 to 12 h before the slaughtering. Before transportation, the catching of animals is followed by crating them in transportation boxes. The transport step varies according to the distance and travel time, depending on the truck type and the driver’s conduction. When it arrives at the slaughterhouse, the load is conducted to a lairage time, preferably in a climatised area. Thus, the unloading and hanging of broilers are finalised with the slaughtering process (Figure 1).

**Figure 2:**
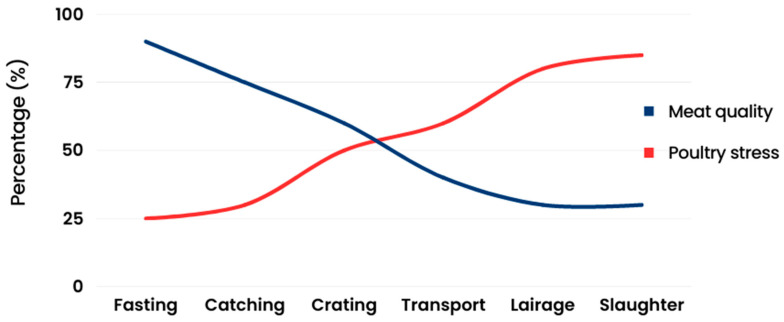
The relationship between stress level and meat quality (adapted from [20,21,22,23,24,25]).

During each step, broilers are subjected to different levels of stress, which are cumulative and represent a reduced welfare level. Also, an increased stress level during the preslaughter operations and a significant reduction in meat quality are expected, mainly after the crating process (Figure 2) [20,21,22,23,24,25].

### 3.1. Fasting

Feed removal before catching, loading, and transporting broilers is a recommended practice for eliminating the contents of the gastrointestinal tract to reduce the risk of faecal contamination of the carcasses during evisceration, as well as of the processing plant and of the operators, ensuring carcass hygiene [26].

Fasting efficiency depends on the feed withdrawal period, which must be long enough to allow for digestive tract cleaning and short enough to avoid the loss of body weight before the slaughter and the subsequent loss of quality in the carcass yield [7]. Aside from this, feed removal before slaughtering minimises the difficulties in meat processing and the consumers’ rejection of processed fatty meat due to its effect on human health [27].

Several authors indicate that the recommended fasting time for broilers before slaughter is 8 to 12 h [28,29,30]. Şengör et al. [31], evaluating different fasting periods (0, 6, 12, and 18 h) before slaughter and their consequences on body weight loss, reported that the most appropriate period was 6 h of fasting, with a body weight loss of 1.89%. A study by Benyi et al. [27] on the fasting period for broilers indicated that 6 to 10 h did not significantly affect body weight. A recent study [32] suggests that fasting for 6 to 10 h dramatically reduces the likelihood of carcass contamination, limiting weight loss in broilers.

Nijdam et al. [32] documented birds’ weight loss after fasting and transport, with results of approximately 0.42% per hour, which was 0.3% more per hour than when the birds were not fasting before transport. If the fasting time is too long (more than 12 h), the body weight of the birds decreases due to dehydration occurring in the muscles [33], resulting in carcass condemnation [10]. In the case of a fasting period of greater than 12 h, the contamination of the canal by faeces increases, since the walls of the intestines become fragile, and the risk of rupture during evisceration tends to increase significantly [34]. In addition, according to Buyse et al. [34], fasting affects several metabolic processes: it causes a change from anabolism to catabolism, from lipogenesis to lipolysis, and a reduced metabolic rate. The increase in fasting time is positively correlated with more significant stress in the animal. This could cause the inadequate acidification of meat, resulting in a flaccid and exudative texture, dark colour, and low lightness and firmness (pH < 5.8) [34,35,36], which is a reason for decomposition, confiscation, and high economic losses.

Further, hot environments can also lead to negative impacts when fasting time is considered. On days with very high temperatures and humidity (above 30 °C and a relative humidity above 80% inside the house) [37], birds decrease or even cease their food consumption during the afternoon or peak heat hours. If applicable, fasting could start later in the evening. The fasting period of birds subjected to hot climatic conditions may be greater than previously defined [38]. Therefore, the first preslaughter operation (fasting) should be planned very carefully (as fasting is the first stressful factor in a succession of other aspects of the same type), considering the consumption time in the entire preslaughter period, to contribute to the reduction of production losses and economic losses in chickens due to death on arrival (DoA).

### 3.2. Catching

The catch is the process by which the birds are captured inside the house through mechanical or automatic methods and deposited in boxes or crates [39,40,41], which are generally made of plastic material. These are later loaded onto a truck and transported to the slaughterhouse. This stage is the most stressogenic in the production of broilers. It could stress the animals and or cause damage to the welfare of the animals [40,42,43].

Among these two methods, the manual capture method is mainly used in the poultry industry. Also, following international animal welfare recommendations [44,45], broilers must be seized and carried by the chest while the birds are upright. This is different on each farm. Broilers, often in groups of five, are commonly captured and carried by the operators with one leg in each hand. Birds are held in an inverted position (head towards the ground) or, less frequently, they are captured by the neck, which is not recommended, as it could cause scratches on the back and thighs and cause suffocation when introducing the birds into the cages [41,46].

This type of management significantly impacts the poultry industry, causing animal stress and injuries. Some studies identified the injury as a significant factor in 30–35% of DoA postmortem examinations [47]. These injuries include trauma, bruising, fractures, dislocations, and liver and head rupture [42]. In a study carried out by Langkabel et al. [47] comparing injuries to the body, legs, and wings after the manual capture of broilers by one or two legs in two weight classes (light: 1.9 kg; heavy: 2.5 kg) and two capture teams (T1 and T2), found that the wings suffered the highest percentage of lesions for both capture groups: for wing lesions, the wing haemorrhages for light animals varied from 0 to 0.04% and those of the heavy animals from 0.32 to 0.48%; wing fractures ranged between 3.57 and 4.74% (light animals) and between 11.62 and 13.97% (heavy animals), with no statistical difference for the capture teams. On the other hand, Kittelssen et al. [8] quantified the number of body broilers with wing fractures before slaughter. Fractures were identified in 7% of DoA cases, comprising fractures of the vertebrae (3.6% of DoA), skull (1.2%), wings (0.5%), and femur/tibia (1.5%). Liver rupture was found in 6.1% of DoA cases, and muscle injury in 1.5%.

To maintain the welfare of the birds, it is necessary to reduce both the physiological stress and the injuries caused at this stage. The temperature of the internal environment of the aviary before the catching is essential for good management in regions with a predominance of high temperatures throughout the year [48,49]. Catching should occur at night during hot weather due to lower temperatures [42]. Likewise, the birds have a reduced visual capacity at night, and as a result, injuries and damages do not occur in the carcass at the time of loading and transport.

### 3.3. Crating and Transport

Broiler transport is currently under increased scrutiny, under which the welfare of the broilers as well as the causes of significant economic and production losses are being scrutinised. During transport, broilers are subjected to several factors that can cause physiological and psychological stress (e.g., distress and pain), such as feed and water deprivation, weather conditions, vibrations, noise, and human–animal interactions [50].

The highest death-on-arrival percentages are found in the loading and transportation stages [7]. At these stages, two essential points need to be considered: (1) the density of birds per box/crate and (2) the microclimatic conditions inside the crate during transport. Stocking density is associated with the increased death on arrival (DoA) of birds at the slaughterhouse. The leading causes of chicken breast bruises, haemorrhages, or fractures are the quick placing of birds through a small opening in the top of the transport boxes and an unregulated capture system, which places the birds in the crates for transportation [41,42,43]. Rocha et al. [50] point out that 90% of the lesions observed during meat inspection are in the preslaughter stages. Previous studies have shown that hand loading generated 8.4% of the wing bruises found in broiler birds [41,47].

The optimum stocking density varies with ambient temperature and bird size [51]. Lower densities would allow more space per bird to sit and be able to regulate its body temperature through adaptations to maintain homeostasis. However, these same gaps among birds could increase the risk of broilers being thrown around, causing physical injury [52]. A high density per cage could reduce transport costs but could result in heat stress [53,54]. Hussnain et al. [54] mentioned that 12 birds per box resulted in a significant losses of carcass and meat properties, and a density of 10 birds per box produced a mortality of 1%. Vieira et al. [55] evaluated the effect of the daily thermal condition considering the number of birds per cage on mortality rates. They reported that five birds per cage during the morning dramatically reduces mortality. During the afternoon, they suggest a density of seven birds per box, considering the increase in mortality due to the temperature of the external environment. Delezie et al. [52] corroborate similar results, indicating that the ideal number of birds per cage during hot and cold days fluctuates between seven and eight.

Weather conditions could influence the microclimate of the cage during transport. High environmental temperatures and high humidity promote changes in bird behaviour to assume environmental changes better [1,56]. Based on this, load wetting is not recommended due to the short effect before the poultry transport [57]. Regarding the thermal condition during transport, through a computational fluid dynamics study, Pinheiro et al. [56] stated that the microclimatic conditions on the truck types, which are not climatised, have the most significant impact on losses and deaths directly linked to thermal stress. In another study, an alternative layout with a central span showed better longitudinal ventilation and wind inside crates when compared with the conventional air-corridor load [58,59]. On the other hand, the same authors observed that the central span model implies more journeys due to the reduced number of crates. This study highlighted the need for studies regarding load layouts for poultry transportation.

This microclimate depends mainly on the density of birds in the transport boxes, the transport distance, and, most importantly, the external environmental conditions [60,61]. According to Merat [62], heat stress in birds is established when the ambient temperature exceeds 25 °C and the cold temperature is below 20 °C, on average. The comfort temperature for fast-growing birds, like broiler chickens, ranges between 22 °C and 25 °C. Birds under heat stress spread out their wings to dissipate sensible heat, open their beaks to dissipate latent heat, lower their heads, and try to make chest contact with the ground to dissipate heat by conduction [55]. Similarly, the sympathetic nervous system (SNS) increases the respiratory rate and catecholamines stimulate a reduction in body temperature [63].

The most appropriate way to transport broilers, reduce thermal stress and production losses, and increase welfare would be to carry them within the thermoneutral zone. This would allow the birds to maintain their body temperature and normalise their metabolic system, keeping the humidity at the appropriate levels [59]. The trucks’ central and rear parts present the highest temperatures during transport, directly related to higher production losses [62,63]. This is mainly due to the difficulty in improving the air quality, which is affected in these regions. Air quality is greatly influenced by reduced air circulation, high humidity, and the heat generated by animals to reduce their body temperature [61], and, in some cases, it is due to an external heat source from the vehicle engine. Soleimani et al. [64] reported 8% mortality in roosters and 4% mortality in 28- to 35-day-old pullets in cages for 3 h at 35 °C and from 65 to 75% relative humidity.

On the other hand, transporting at low temperatures could cause hypothermia, affecting broilers’ slaughter performance and welfare [62,65]. Cold stress has been reported to activate the hypothalamic–pituitary–thyroid axis of the bird and increase the corticosterone concentration [65]. When transported to the slaughterhouse, broilers’ high dead-on-arrival (DoA) percentages increase when the external temperature is less than 18 °C and higher than 30 °C [61,66]. Tao et al. [67] documented that the exposure of 46-day-old broilers to 35 °C with a high humidity and an air velocity of 0.2 m s^−1^ caused hyperthermia within 1.5 to 4 h. While increasing the air velocity to 0.7 m s^−1^ or reducing the humidity only increased rectal temperature by approximately 2 °C and was not lethal (Table 1).

Another point that significantly influences the welfare of broilers is the distance and duration of transport, which subject the broilers to stressful conditions. Various authors indicated that durations greater than 2 h could cause damage and mortality in the broilers inside the truck [68,69]. However, several studies, such as Oba et al. [69] and Aral et al. [70], have reported that at shorter distances, the proportions of dead animals are lower than at long distances. Warriss et al. [71] found that broiler mortality was 0.156 and 0.283% for trips of less than 4 and of more than 9 h, respectively. These results could be corroborated by Bianchi et al. [72], who indicated that the mortality of birds for journeys shorter than 3.5 h, between 3.5 and 5 h, and more prolonged than 5 h was 0.24, 0.41, and 0.45%, respectively. Besides, Vecerek et al. [73] found mortality rates of 0.146 and 0.862% for travel distances of less than 50 km and of greater than 300 km, respectively.

We could postulate that weather is decisive in reducing injuries such as bruises, haemorrhages, fractures, and death. The density of birds per cage and the temperature are essential factors. They could generate high stress levels in broiler chickens, increasing their respiratory rates due to their inability to reduce their temperatures at high thermal levels, resulting from temperature fluctuations inside and outside the cage during transport. Therefore, the opinion of the EFSA [74] could be considered, who suggested that the number of birds in the cages/crates should be limited (i.e., low density) when environmental temperatures are higher than 22 °C. Similarly, in cold seasons, a higher density could be considered, since increasing the density of birds during the trip could have a beneficial effect. The thermal concentration inside the cage is essential for the birds during cold seasons. In this condition, the metabolic body heat could warm the air and reduce the risk of death from hypothermia [73,75,76].

### 3.4. Lairage

After transport, the birds go through the waiting process, a period between the vehicle’s arrival and the unloading boxes, where the trucks must be parked in an air-conditioned environment that allows the birds to regain their thermal comfort [50,75,76]. Lairage places could be open or closed. However, it is recommended that the system be covered and away from sources of solar radiation and humidity, since the temperature control systems could consist of fans, exhausters, and foggers [63].

The fans and exhaust fans must be located strategically, removing the heat from inside the boxes. Therefore, the fans are not expected to be placed on the trucks, since they could contribute to a hot air rise within the truck [50]. Foggers must be used with fans and exhaust fans at a low relative humidity, allowing humid air to move and reducing the truck’s temperature [77]. Their use must be controlled, since in conditions of high humidity or cold they could become a stress factor or hinder the thermal control of the animals. Therefore, the conditions to which birds are subjected during lairage must be conducive and stress-free and prevent infections among the birds from minimising carcass condemnation.

This lairage procedure is of great importance and should not be prolonged, since the birds have a low availability of active metabolic energy, otherwise physiological changes, weight loss, and the more significant presence of bruises due to the sequence of stressors that birds were subjected to, such as fasting, catching, and transport, may occur [10,78,79]. Similarly, this process could help reduce deaths and costs and improve the final product quality [80]. Prior studies evaluated the lairage time until slaughter, where recommendations vary between 1 and 2 h [78,81]. However, the time interval may be planned into a logistics program, depending on the variable of interest (e.g., distance, season, thermal conditions during transport, and crating densities) [55,79,80,81,82,83,84].

A factor that could be used to help control the time until unloading is the transport distance. Over short distances, the thermal stress of the animals could be reversible with acclimatisation, if the birds are able to remain longer in lairage for recovery; since long trips can take the animal to a critical state, reaching irreversibility caused by the animal’s energetic depletion, short lairage periods are recommendable [79]. This could result in darker meat [85], which the consumers accept less. Another critical variable is the season of the year. Vieira et al. [82] observed differences between the mortality rates during lairage periods over the seasons. The same authors stated that the death on arrival rate is higher during summer than in other seasons. However, the mortality rate was reduced when the broilers were subjected to long lairage periods (3 to 4 h). A similar pattern was observed during harsh periods (air temperature above 22 °C—[83,84]). The authors found higher rates of poultry mortality when lairage periods below 2 h were adopted in hot periods of the day [86,87]. Thus, climatised slaughterhouses and the lairage period can be considered relevant points for monitoring the welfare of birds, allowing studies that make it possible to offer greater thermal comfort to animals and lower preslaughter losses.

### 3.5. Unloading

During loading, transport, and lairage, broiler deaths due to stress could occur; this fact is known as “death on arrival” (DoA) [88]. The DoA rate could be obtained after unloading the trucks and opening the transport boxes, which allows animals to be counted, and values obtained (%). Constantly trained employees must unload and transport the boxes with birds to the hanging area, which must be carried out calmly and peacefully. For this step to be carried out efficiently, it is recommended to use mobile platforms that help allow easy access to the vehicle body, allowing handling without sudden movements [77] and reducing the risk of injuries and fractures. The animals must be removed from the boxes and hung on the rail quickly and calmly. The birds must not remain loose in this environment [85,89]. According to the same authors, the dead birds are separated and discarded.

### 3.6. Hang at the Slaughter Line

Hanging is a procedure that aims to send the birds coming from the unloading of trucks to slaughter [90]. The conveyor, i.e., the equipment responsible for this process, has clips (hooks) that allow the fitting of the broilers’ legs, allowing them to be fastened upside down and in an automated way, forwarding the broilers to the stunning vat, one of the main methods commonly used [91]. This process makes the broiler slaughter system extremely fast and convenient.

Some issues affect the welfare of handling birds during the pre-stunning periods [92]. The main precautions to be taken are linked to handling animals during hanging, seeking to avoid injuries, and factors related to the birds’ reactions, such as fear, pain in the legs and shins, and stress [93]. Broilers have 21 nociceptors on the lateral surface of their legs, which are responsible for the sensation of pain while hanging, so it is recommended that the animal be stunned within a maximum period of 12 to 60 s after hanging [90].

The correct hanging of the animals is very relevant to the desensitisation of the birds. Animals positioned incorrectly may not be induced to unconsciousness adequately and painlessly, as the electrical circuit must be closed with the bird’s contact with the conveyor and vat. Another point to be observed related to the hanging and stunning procedures is the fact that stressed or injured animals show more significant movement in the attempt to escape, which may cause contact of the wings with the electrified water, causing shocks and pain [94] or even fractures, before stunning [49].

In addition, other factors could help the process to occur efficiently. Examples are low light levels in the hanging environment; no unnecessary accumulation or transit of people in the place; the use of chest support structures (parapets); and the proper handling of animals. These are ways to increase the welfare of broilers and induce them to the highest level of tranquillity, to benefit the entire system [95]. The reduction of unevenness, curves, and obstacles is also recommended in this system.

### 3.7. Stunning

It is understood that birds are sentient; thus, the stunning induces unconsciousness in the preslaughter period to reduce the possibility of understanding the suffering during bleeding (neck cutting) [93]. Seeking to align such slaughtering practices and the need to improve animal welfare at this time, stunning methods have been updated over time, always aiming at maximum welfare, which allows the consumer to be offered a better-quality product. There are several stunning methods available for birds. The main ones are based on electricity, physical procedures, or a controlled atmosphere. They all require authorisation from current legislation before being used in supervised slaughterhouses.

The primary method used is electronarcosis in immersion vats. This is due to its reduced cost when compared to other processes. This practice consists of hanging the birds by the legs on metal hooks connected to a moving wheel. The animal has its head immersed in a reservoir with electrified water, resulting in the immediate loss of consciousness due to the passage of electric current through the bird’s brain, depolarising neurons and temporarily preventing the transmission of stimuli [77]. Numerous studies indicated that a current of 120 milliamps (mA) per bird is required to produce the immediate loss of sensitivity to pain and unconsciousness [93,96].

Several factors could impair the efficiency of electronarcosis, such as inhomogeneous batches (in size and weight) and different current levels, waveforms, and frequencies, which could be affected by water hygiene [96,97], making the method complex. The immersion method could directly affect the quality of the final product. At high electrical loads, severe muscle contractions, blood spatter, and fractures might occur [97,98]. However, this does not indicate the inefficiency of the stunning method or the reduction of bird welfare.

Another form of stunning that could be used is the method of individual electronarcosis, namely, electrodes on the head, where the birds are placed upside down in a cone, and two electrodes are placed on the sides of the brain [97]. Like the previous method, the method of electrodes from the head to the cloaca contains two electrodes, each positioned at each of the organs mentioned before. The circuit is closed to allow the passage of electric current [98]. Also, the method of controlled atmosphere models could be used to induce unconsciousness. In this method, different combinations of gases are used. The most frequently used is carbon dioxide (CO_2_), in which birds are taken to a greenhouse and subjected to two gas exposure phases. In one of the phases, birds are exposed to a CO_2_ concentration of 40% of the total gases, causing gradual unconsciousness, and in another, they are subjected to 80 to 90% of other CO_2_ gas concentration, confirming unconsciousness until brain death at the time of sticking [94]. However, this procedure does not present immediate stunning, which can harm animal welfare in the preslaughter stage if not conducted correctly.

As concluded by Berg and Raj [99], it is understood that studies should be continually seeking the improvement of existing methods, with a focus on reducing their disadvantages and the creation of new technologies that would allow an effective and rapid process of inducing unconsciousness, the reduction of costs and the commercialisation of better-quality products.

### 3.8. Slaughtering

Currently, different forms of slaughtering methods are used. This diversity is directly linked to religious and cultural factors [100], as well as animal welfare. Considering the above points, each country has legislation describing the processes to be carried out.

The types of slaughter can be classified into conventional or religious. The traditional method is based on stunning and bleeding animals, emphasising welfare, and seeking to reduce animal suffering, pain, and trauma. However, religious practices are based on cutting the neck with the aid of a sharp knife, with or without prior stunning [100], following the principles of each religion. Following religious precepts seeks to respect and meet the demands of the Muslim and Jewish communities, while both constitute their diets based on the teachings of their sacred books. Furthermore, applying these methods aims to respect the population’s right to religious freedom.

The two types of religious slaughter used on an industrial scale are Halal (Muslim people) and Kosher (Jewish people). The Halal method consists of restraining the live animal and cutting the neck for bleeding with a sharp blade/knife. The slaughter must be done quickly, in Allah’s name, and be carried out by a devout Muslim [101]. In general, the Kosher slaughter method is more rigorous about animal selection, the implementation of techniques, and the postmortem evaluations of the viscera compared to the Halal method, which is considered more flexible [101,102,103,104,105,106,107,108,109]. The Kosher method is a slaughter technique that is based on cutting the neck (trachea, oesophagus, carotid arteries, and jugular veins) of the animal with an extremely sharp knife, performed by a Jewish devotee with specific training, practising the due rituals, strictly following all the rules described by the religion [103,107]. Otherwise, the carcass is not considered Kosher and cannot be consumed by Jews. In this slaughter model, no form of prior stunning is allowed [104]. The consumption of blood in the Jewish religion is also restricted; for this reason, the arteries and veins are removed, go through a process of salting (for about 1 h), washing (three times), and draining for a period of up to 72 h after slaughter [105,108].

Historically, the slaughter was carried out in this way, following the description of the Prophet [108]. The point of most significant divergence in practice is related to stunning because, in the holy book, there is no description of this technique while emphasising the care that must be adopted. To not subject the animal to suffering, there is permission (but not a consensus) about carrying out the stunning, as long as it is temporary and does not lead to death [106,107,108,109,110,111]. Another point required is that there is no interference in the bloodletting of the animal, since the holy book restricts the consumption of blood [106].

In all cases, bleeding is used to slaughter animals in slaughterhouses. The technique takes the chicken to a condition of central nervous system ischemia and the cessation of its activity, generally after draining 50% of the body’s total blood [106]. This process is essential to maintain the quality and durability of the carcass, the flavour, and the technological values of the meat that will be offered to consumers, since it can hurt the product’s acceptability, appearance, and shelf life [110,111].

## 4. Looking Forward: The Concept and Practice of Animal Logistics

As discussed above, animal stress and suffering are considerable at all the stages of preslaughter operations. While the industry focuses on yield and carcass quality, animal welfare is neglected. The scenario is even worse, considering the poor or absent logistics in planning these stages. There is an urgent need for logistics to be professionally implemented in companies, as described below.

Logistics, in real terms, include all the processes enabling the movement of things, either living or non-living, from one place to another [112]. The success of every sector, including animal production, is fundamentally determined by the logistics implemented, as they are an essential component [113,114]. The world has seen significant advancements in transportation and logistics [114], which can be attributed to the various designs of the vehicles used for transportation.

However, these advancements have yet to impact the livestock industry, particularly animal logistics, significantly. The stress that animals experience during transportation remains a critical issue that needs to be addressed. Surprisingly, much of the effort related to logistics focuses on the effects of transportation and logistics management on the food supply chain and its sustainability [114,115], with little consideration for the welfare of animals, which play a key role in the food supply chain.

Logistics in the agricultural sector is understandably more complex than other sectors [114]. Despite these complexities, integrating technology for real-time monitoring in the sector [114] could significantly reduce the challenges faced in animal logistics. The vast amount of data generated daily in animal logistics could be leveraged to improve animals’ conditions during transit, ensuring better welfare and efficiency. This technology would also enable stakeholders to provide animals with comfort, including freedom from distress, pain, injuries, and illnesses [116] during transportation.

Effective and qualitative transportation planning and truck monitoring are crucial for ensuring the welfare of transported animals. Proper planning enables farmers and transporters to manage transport time and minimise the number of efficient pick-up stops when transporting animals to slaughterhouses [116]. According to the study by Frisk et al. [117], the duration of the planning phase significantly impacts animal welfare. They found that the length of the planning window influences transportation time, the total distance covered, and the duration the truck spends on the road before reaching the slaughterhouse. Their study concluded that reducing the number of pick-up stops and the transport time can significantly enhance animal welfare during transportation. Therefore, meticulous transportation planning is essential for improving the conditions under which animals are transported.

In addition to the planning time and the number of pick-up stops, optimisation modelling that maintains animal welfare is crucial to the logistics plan’s success. The challenge with this type of optimisation is that most of its applications are with inanimate cargo. The transport of animals is considered live cargo and poses a challenge, especially considering the many variables to be included in the model. A successful application was described by Frisk et al. [117], who developed an optimisation model for one year’s worth of cattle transport data, considering the length of time, short and long distances, the reduction of stops during the journey, and the limitation of transport time on each stretch. The authors found that optimisation modelling, reducing stoppage time, or reducing transport time improves animal welfare during transport. However, there is still much to be done, such as including more variables, especially thermal conditions, which are crucial for hot weather and in times of climate emergency.

Implementing close monitoring programs in animal logistics is essential to ensure optimal animal environments during transportation to and from farms. Precision livestock farming technology, already in use in livestock buildings, should be integrated into animal logistics. This technology will assist truck drivers in minimising stress on the animals, thereby preventing the adverse effects on their physiological and behavioural responses [116] during transit.

## 5. Conclusions

Thermal challenges and live load logistics are key issues for poultry preslaughter operations. Broilers often suffer from heat stress from catch to slaughter, combined with weak or no optimisation planning within a logistics monitoring programme. The same was observed in scientific research. Several shortcomings remain regarding the welfare of poultry during transport. Some strategies are recommended to improve this scenario.

During catching and crating, the animals must be kept in a shaded area without direct exposure to the sun. Transport should preferably take place at night. If this is not possible, the route should be planned considering the weather conditions, and the cargo should be transported in an adapted lorry, aiming at a minimum thermal comfort for the broilers. The lairage, another step on which few studies are conducted, must be arranged for a short period (1–2 h) in a climatised area. However, distance and external thermal conditions are key points when planning the lairage area. Finally, the slaughter environment should be thermally suitable for broilers and workers to reduce stress and losses. A professional preslaughter operation considers logistical planning with good optimisation modelling, including the variables of the thermal environment, time, distance, and people involved.

## Figures and Tables

**Table 1:** The thermal zones for poultry and their relationship with death-on-arrival (DOA) risks.

Temperature (°C)	Relative Humidity (%)	Wind Speed (m s^−1^)	DOA Risk *
<18	<60 or >75	>1.0	+
18–22	65–75	0.3–0.7	−
22–25	65–75	0.3–0.7	+ −
>25	<60 or >75	<0.3	+

* + high risk of DOA; + − average level of DOA; − low risk of DOA. Thermal zones of the external environment during transportation. These values do not consider the thermal gradient inside the load.

## Data Availability

Not applicable.
